# Social jetlag and sleep habits in children and adolescents: Associations with autonomy (bedtime setting and electronics curfew) and electronic media use before sleep

**DOI:** 10.1080/07420528.2024.2444675

**Published:** 2025-01-06

**Authors:** Gaby Illingworth, Tanya Manchanda, Simona Skripkauskaite, Mina Fazel, Felicity Waite

**Affiliations:** a Department of Psychiatry, University of Oxford, Oxford, UK; b Sir Jules Thorn Sleep and Circadian Neuroscience Institute, University of Oxford, Oxford, UK; c Department of Experimental Psychology, University of Oxford, Oxford, UK; d Oxford Health NHS Foundation Trust, Oxford, UK

**Keywords:** Social jetlag, sleep habits, bedtime, electronic media, social media, video gaming, children, adolescents

## Abstract

For young people attending school, social jetlag (SJL) refers to discrepancy in sleep/wake timing between school days and weekends. This study investigated SJL in school-aged children and adolescents in England and whether this is associated with age, gender, and sleep habits including bedtimes and electronic media use. Students (school y 5–13; typical age 9–18 y) completed the 2021 OxWell Student Survey. In total 19,760 participants (55% female) reported on sleep/wake timing, rules concerning bedtime setting on school night/weekend, electronic media curfew, and frequency of social media use and video gaming before sleep intention. The mean SJL was 1 h 53 min (*SD* = 1 h 7 min) and peaked at 2 h 7 min at age 15. Multiple regression analysis revealed SJL was positively associated with age and being male was associated with slightly lower SJL than being female. After controlling for age and gender, weekend bedtime setting (β = 0.21), frequency of social media use before sleep (β = 0.16) and video gaming before sleep (β = 0.12) were the strongest predictors of SJL. Findings suggest that household rules regarding weekend bedtimes and less electronic media use before sleep may be connected with lower SJL as well as more regular sleep timing across the whole week.

## Introduction

Social jetlag is used to describe the misalignment of social and biological time, demonstrated by a discrepancy in sleep timing and activity on “work” and “free” days (Wittmann et al. 2006). Sleep on free days is thought to better reflect an individual’s natural sleep timing, while sleep during the working week is constrained, reflecting external or social time (Vetter 2020). Social schedules may impact on an individual’s daily behaviour, so that they are active and asleep outside their circadian window (i.e. window of biological sleep timing provided by the circadian clock). For children and adolescents attending school, social jetlag is defined as the discrepancy in sleep/wake timing between school days and weekends. High social jetlag is demonstrated by a pattern that shows much variability between school day and weekend sleep. In adolescence, social jetlag has been found to be associated with irritable mood (Tamura et al. 2022), anxious symptoms (Mathew et al. 2019), decreased academic and cognitive performance (Díaz-Morales and Escribano 2015; Smarr and Schirmer 2018; Tamura et al. 2022), somatic complaints and attention problems (Zhu et al. 2023), and excessive alcohol consumption (Haynie et al. 2018). In addition, social jetlag has been identified as a risk factor for metabolic disorders and obesity (Roenneberg et al. 2012, 2019). These findings suggest it may be important to examine social jetlag, or irregular sleep timing – over and above other aspects of sleep, such as late bedtimes and short sleep duration – when considering possible challenges to a healthy lifestyle.

Social jetlag increases from childhood to adolescence, and is most acute during mid adolescence before declining (Randler et al. 2019; Roenneberg et al. 2012, 2019), corresponding with what is termed the biological “phase delay” that pushes bedtimes and waketimes later in puberty (Crowley et al. 2018). Individuals vary in how their circadian system synchronizes with zeitgebers (environmental time givers); for example, the 24-hour light-dark cycle. When viewed as a biological construct, these differences in “phase of entrainment” are known as chronotype (Ghotbi et al. 2020; Roenneberg et al. 2019). Adolescents tend to be later chronotypes than other age groups (Roenneberg et al. 2004) and may have school schedules that contradict their chronotype, with sleep timing constrained in part by early morning schedules (Carvalho-Mendes et al. 2020; Ziporyn et al. 2022). Accordingly, young people may struggle to sleep early enough on a school night and also struggle to wake up when required for the start of the school day. In contrast, with fewer demands of the social clock, individuals are more likely to sleep to their own timing on weekends, if no additional extra-curricular or work constraints are placed on them. Young people may “compensate” for insufficient sleep during the school week, with an extended sleep duration and later wake times at the weekends (Gradisar et al. 2011; Touitou 2013). Findings regarding gender differences in social jetlag are equivocal. No significant gender differences in social jetlag have been found in a cross-sectional sample from infancy to 25 y (Randler et al. 2019), but social jetlag has been found to be greater in school-aged boys than girls in some studies (Chandrakar 2017; Díaz-Morales and Escribano 2015) as well as in school-aged girls than boys in other studies (Shinto et al. 2022).

Given that the timing of sleep matters, it is important to consider whether changes to evening routines could reduce social jetlag. Parents and caregivers may be influential in adolescents’ sleep patterns, through setting bedtimes (Carskadon 2011) and supporting regular bedtimes and waketimes. One of the defining features of adolescence is the desire for, and acquisition of, greater autonomy (Blakemore 2019; Zimmer‐Gembeck and Collins 2006). This striving for behavioural autonomy may conflict with parental/household rules and have implications for sleep timing. As children move into adolescence, the proportion of parents setting bedtimes reduces (Carskadon 2011; Tashjian et al. 2019). A study in Australia found that 17.5% of adolescents, aged 13–18 y, reported a parent-set bedtime to be the main determinant of when they went to bed on a school night (Short et al. 2011).

Research around the influence of bedtime setting has largely focused on connections between who sets young people’s bedtimes, and their reported bedtimes, sleep duration and sleep quality (Adam et al. 2007; Bartel et al. 2015; Buxton et al. 2015; Gangwisch et al. 2010; Khor et al. 2021; Peltz et al. 2020; Pyper et al. 2017; Short et al. 2011, 2019; Tashjian et al. 2019). Parent-set bedtimes have been reported as a protective factor and found to relate to longer sleep duration (Bartel et al. 2015; Khor et al. 2021; Peltz et al. 2020). Parental rules around bedtimes have been found to relate to adolescents having earlier bedtimes and greater total sleep on school nights (Adam et al. 2007; Short et al. 2011, 2019; Tashjian et al. 2019) and at the weekend (Short et al. 2019; Tashjian et al. 2019) compared to when adolescents set their own bedtimes.

Most studies investigating parental rule setting for electronic devices and sleep have focused on the timing and duration of sleep rather than its effects in relation to social jetlag. A systematic review and meta-analysis concluded that earlier bedtimes and longer sleep duration were more likely when parental rules were present for electronic device use (Khor et al. 2021). However, enforcement of rules related to electronic media use before bedtime did not predict longer sleep duration in a sample of 14–17-year-olds in the US (Peltz et al. 2020). Not only frequency of use but timing may be influential for sleep; in other words, it is more likely to have a negative impact if screen use behaviour is before sleep rather than during the daytime. Nighttime social media use has been shown to be associated with later bedtimes and shorter sleep duration in a small sample of 12–18-year-olds in the UK (Scott and Woods 2018). The more frequently adolescents (mean age: 16.9 y) played video games during the week in the hour before going to bed with the intention to sleep, the later were their school day and weekend bedtimes (Pieters et al. 2014). A Swedish study investigated electronic media use and social jetlag with adolescents, and found that the frequency of nighttime texting was associated with social jetlag in 13–15-year-olds (Hena and Garmy 2020).

Discussions around bedtime and when to stop using electronic media at night are likely to be taking place in many households. Exploring the determinants of sleep timing could help establish promising areas for intervention. However, there is a dearth of research examining associations between social jetlag and rules around bedtime setting, rules around electronic media use, as well as the frequency of electronic media use before sleep. To our knowledge, it is yet to be investigated whether young people setting their own bedtime on school nights and/or at the weekend and having more control over their electronic media use is associated with social jetlag. This information could help inform family decisions regarding bedtime setting and electronic media use before sleep, with the potential to offer modifiable actions to lower social jetlag in young people. The aim of this study was to (1) establish the amount of social jetlag experienced by school-aged children and adolescents in a UK sample and whether this is associated with age and binary gender; (2) identify whether rules concerning bedtime and electronic media use are associated with social jetlag; and (3) explore whether the frequency of social media use and/or video gaming before sleep intention are associated with social jetlag in school-aged children and adolescents. We expected to find that social jetlag was positively associated with age, while no predictions were made regarding gender. Based on previous findings regarding sleep timing and sleep behaviours, specifically bedtimes and sleep duration, we predicted that rules around bedtime setting as well as household rules around electronic media use, would be associated with lower levels of social jetlag. Furthermore, we hypothesised that high frequency of social media use and video gaming before sleep would both be related to higher levels of social jetlag.

## Methods

### Design and Procedure

The OxWell Student Survey is a repeated cross-sectional survey measuring the mental health and wellbeing of school-aged children and young people in England, as described in the study protocol (Mansfield et al. 2021). Students attending primary schools (school y 5–6; typical age 9–11 y) and secondary schools (school y 7–13; typical age 11–18 y) – including state-maintained schools, academies and independent schools – as well as further education colleges, were eligible to participate in 2021. However, respondents from the further education college that took part were excluded from this study as it was considered that these students’ weekly schedules may not reflect a typical school schedule, with potentially greater flexibility and possibly fewer early lesson start times.

Schools were recruited with support from local authorities (administrative body in local government) located in four counties in England: Oxfordshire, Berkshire, Buckinghamshire and Merseyside. Students were invited to take part through their school. A parental opt-out process was used for those under 16 y. Online assent/consent was provided by those under 16 y/16 y and over. The study was approved by the University of Oxford Research Ethics Committee (Ref: R62366).

### Measures

The OxWell Student Survey variable guides can be accessed from the Open Science Framework (OSF) website (https://osf.io/sekhr/).

#### Social Jetlag

Social jetlag was examined and calculated from sleep timing measures: “sleep onset latency,” “try to sleep time,” and “wake time” items adapted from the Munich Chronotype Questionnaire (MCTQ) (Roenneberg et al. 2007) and the School Sleep Habits Survey (Wolfson and Carskadon 1998; Wolfson et al. 2003), (Sleep for Science webpage: http://sleepforscience.org).

Sleep onset latency (SOL) was measured by asking participants: “How long do you usually take to fall asleep?” and rated using a sliding scale including five category labels (anchored at “0 min” and at 30-min intervals to “120+ min (2 hours or more)”). Other items, as well as measures calculated from these, captured sleep patterns on school nights and on weekends. For instance, “Try to sleep time” was informed by “What time do you usually try to fall asleep on a school night/at the weekend?” rated using a sliding scale including five category labels at 2-hourly intervals (ranging from “6pm” to “2am or later”). “Wake time” was devised from “What time do you usually wake up on a school day/at the weekend?” rated using a sliding scale including five category labels at 2-hourly intervals (ranging from “5am” to “1pm or later”). Sleep duration was calculated by the difference between “sleep onset time” (try to sleep time plus SOL) and wake time. A total of 184/160 cases with sleep durations (<3 hours or >12 hours) on a school night and 189/103 cases with sleep durations (<3 hours or >14 hours) at the weekend were considered potentially implausible and thus excluded. Sleep duration is operationalised and included as part of the formula used to calculate midsleep times and subsequently social jetlag (Roenneberg et al. 2012). The midpoint of sleep was calculated based on the halfway point of the clock time between sleep onset and wake.

Social jetlag (SJL) was calculated as the absolute difference between the midpoint of sleep at the weekend (MSF) and the midpoint of sleep on school nights (MSW): SJL = |MSF – MSW|. Actual social jetlag was calculated as the actual difference between the midpoint of sleep at the weekend and the midpoint of sleep on school nights: SJLact = MSF – MSW. This provides a relative value and information on negative social jetlag in cases when midsleep times on school days are later than on weekends (Roenneberg et al. 2012, 2019; Wittmann et al. 2006).

### Predictor Variables

#### Demographics

Age was measured in full years. Gender was measured in Year 5–7 as boy, girl or prefer not to answer and in Year 8–13 as male, female, other/prefer not to answer. Gender was included as a binary variable (female, male) and treated as missing data if a response was provided as other/prefer not to answer or was missing. In the context of a lack of gender diverse reference points in the literature, we did not include gender diverse individuals (as captured in “other” or potentially in the “prefer not to answer” category) in this analysis. Instead, data are included in Table S1 in supplementary material so as to provide a preliminary understanding of this sample’s characteristics and social jetlag.

#### Autonomy (Bedtime and Electronics)

Bedtime setting was measured separately for school nights and the weekend by asking: “On a school night (when you have lessons the next day), who usually sets your bedtime?” and “On a weekend night (no lessons the next day), who usually sets your bedtime?.” The response options were: “Yourself” or “Parent/carer/guardian/other family member/other.” A school night binary variable and a weekend night binary variable were then created: participants who set their own bedtime (self), and those whose bedtime was set by another (other). Whether participants had an electronic media curfew was based on: “On school nights (when you have lessons the next day), do you have a rule or set time in your house about when you are supposed to turn off or put away computers, phones or other electronics?.” Response options were binary: “yes” or “no.”

#### Electronic Media Use

Social media use and video gaming before sleep intention were measured by two items: “How often do you use social media (e.g. Tik-tok, Instagram) in the hour before you intend to go to sleep?” and “How often do you play video games in the hour before you intend to go to sleep (including games on consoles, computer, tablet, mobile phone or other portable gaming device)?.” The response options were recoded as: 0 = Never, 1 = Rarely (1–2 times a month), 2 = Sometimes (1–2 times a week), 3 = Often (3–4 times a week), 4 = Daily.

### Analysis

Full details of inclusion criteria can be viewed in the pre-registration for this analysis (Illingworth et al. 2024). The data were checked for standard statistical assumptions (e.g. normality of continuous variables, independence of observations, etc.). Means and standard deviations (SDs) are reported for continuous variables and frequencies and percentages for categorical variables. Descriptive statistics are provided for the amount of social jetlag experienced by age in years and by gender. Gender differences in social jetlag were investigated using a *t*-test.

Multiple regression analysis was used to answer subsequent research questions with social jetlag as the continuous outcome variable included in all models. Analyses were run separately for social jetlag and actual social jetlag. The first model examined the predictive contribution of age and gender to social jetlag; Model 1: age (continuous predictor: age in years), gender (categorical predictor: female/male). We then examined the predictive contribution of bedtime rules, electronic media curfew, social media use before sleep, and video gaming before sleep to social jetlag across partially adjusted models, Models 2 to 4. All models included age and gender as covariates with predictors listed for each model as follows. Model 2: bedtime rules on a school night, and bedtime rules on a weekend night (categorical predictors: other/self). Model 3: electronic media curfew (categorical predictor: yes/no). Model 4: social media use before sleep, and video gaming before sleep (continuous predictors: 0–4). All predictors were included in the final fully adjusted model, Model 5: bedtime rules on a school night, bedtime rules on a weekend night, electronic media curfew, social media use before sleep, and video gaming before sleep. Standardised beta coefficients (β) are used to interpret regression models. All analyses were conducted using R (version 4.3.2; The R Foundation for Statistical Computing). Due to the large sample size, a *p*-value < 0.001 was interpreted as indicating statistical significance.

## Results

### Participants

A total of 30 115 participants accessed the survey and met initial data inclusion criteria (Illingworth et al. 2024). Participants aged 19 y and above, or below 9-y-old due to few responses, were excluded as were those who did not provide their age (*n* = 274). Of the 29 841 participants aged between 9 and 18 y, 6,342 did not provide data that enabled social jetlag to be calculated (i.e. did not respond to all necessary school and weekend sleep timing variables). Of the remaining sample (*n* = 23,499), 1,205 students did not indicate a binary gender (female, male). A descriptive comparison of included and excluded participants regarding gender can be found in Table S1 in supplementary material. The final sample used for all analyses included 19 760 participants (55.4% female) without further missing predictor information concerning autonomy (rules on bedtime setting and electronic media) and electronic media use (social media and video gaming) before sleep, while 2,534 participants missing one or more predictor variables were excluded. Participant characteristics are provided in Table 1 and for included and excluded participants in Table S2 in supplementary material.

**Table 1.** Sample characteristics for students who provided social jetlag with all predictor variables.

	Social jetlag and all predictors
	(*n* = 19,760)
Age (years), n (%)	
9	406 (2.1%)
10	2598 (13.1%)
11	2699 (13.7%)
12	3524 (17.8%)
13	3594 (18.2%)
14	3347 (16.9%)
15	2312 (11.7%)
16	353 (1.8%)
17	841 (4.3%)
18	86 (0.4%)
Mean (SD)	12.7 (1.94)
Gender, n (%)	
Female	10946 (55.4%)
Male	8814 (44.6%)
Social jetlag (h)	
Mean (SD)	1.88 (1.12)
Actual social jetlag (h)	
Mean (SD)	1.85 (1.17)
School bedtime setting, n (%)	
Bedtime set by other	10425 (52.8%)
Bedtime set by self	9335 (47.2%)
Weekend bedtime setting, n (%)	
Bedtime set by other	5397 (27.3%)
Bedtime set by self	14363 (72.7%)
Electronic media curfew, n (%)	
Yes	10202 (51.6%)
No	9558 (48.4%)
Social media use before sleep, n (%)	
Never	2960 (15.0%)
Rarely	1707 (8.6%)
Sometimes	2290 (11.6%)
Often	3064 (15.5%)
Daily	9739 (49.3%)
Mean (SD)	2.75 (1.50)
Video gaming before sleep, n (%)	
Never	3745 (19.0%)
Rarely	3511 (17.8%)
Sometimes	3505 (17.7%)
Often	3588 (18.2%)
Daily	5411 (27.4%)
Mean (SD)	2.17 (1.48)

Social jetlag is reported in decimal hours. Electronic media before sleep is scored 0–4.

### Distribution of Social Jetlag, and Social Jetlag by Age and Gender

**Figure 1.**
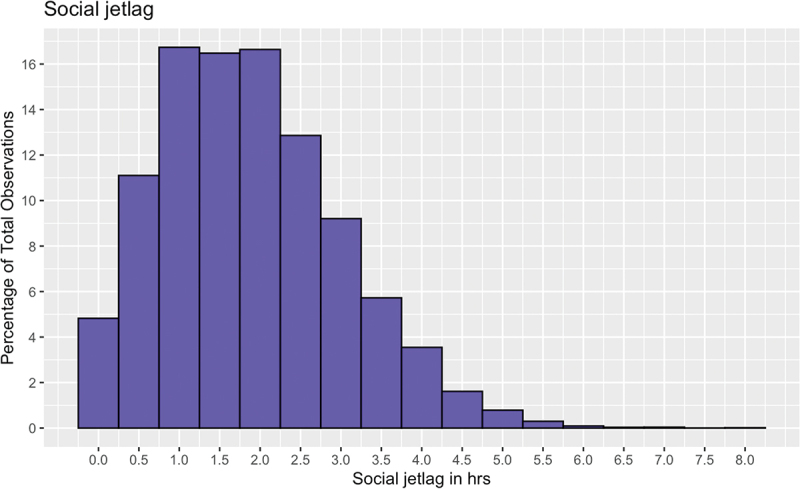
Distribution of social jetlag. The distribution of social jetlag is based on half-hourly bins.

**Figure 2.**
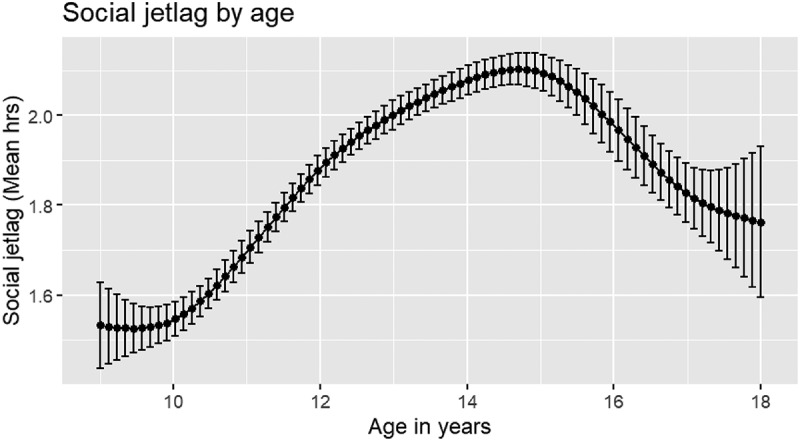
Social jetlag by age.

**Figure 3.**
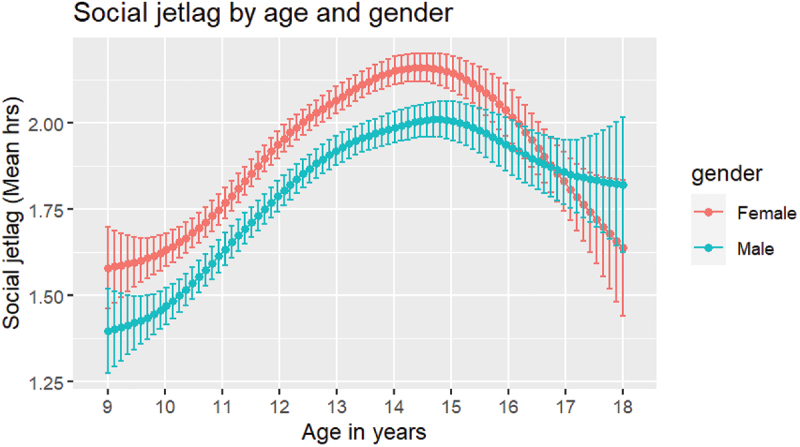
Social jetlag by age and gender.

The amount of social jetlag experienced by students is shown in Figure 1. The majority 19 728 students (99.8%), demonstrated social jetlag (difference in midpoint of sleep at weekend and on school night = >0 h), while 32 students experienced no social jetlag (midpoint of sleep at weekend and on school night are the same). When considering actual social jetlag, only 649 students (3.3%) reported social jetlag that meant midsleep at the weekend was earlier than midsleep on school nights (i.e. negative social jetlag). The mean social jetlag was 1.88 h (*SD* = 1.12 h) while actual social jetlag was approximately the same at 1.85 h (*SD* = 1.17 h). For social jetlag by age in years and gender, see Table 2, Figures 2 and 3. The smallest social jetlag was experienced at age 10 (*M* = 1.53 h, *SD* = 1.18 h; actual social jetlag: *M* = 1.46 h, *SD* = 1.26 h) and the highest at age 15 (*M* = 2.11 h, *SD* = 1.05 h; actual social jetlag: *M* = 2.09 h, *SD* = 1.09 h). Females demonstrated higher social jetlag than males (0.15 h; 95% CI, 0.12–0.18 h, *t*(18616) = 9.29, *p* < 0.001; *d* = 0.13) as well as higher actual social jetlag than males (0.17 h; 95% CI, 0.14–0.20 h, *t* (18420) = 10.13, *p* < 0.001; *d* = 0.15).

**Table 2.** Descriptive statistics for social jetlag by age, gender, bedtime setting, electronic media curfew, and frequency of electronic media use before sleep (*n* = 19,760).

		Social jetlag (h)	Actual social jetlag (h)
	*n*	Mean (*SD*)	Mean (*SD*)
Age (years)			
9	406	1.58 (1.15)	1.48 (1.28)
10	2598	1.53 (1.18)	1.46 (1.26)
11	2699	1.70 (1.12)	1.65 (1.19)
12	3524	1.89 (1.12)	1.87 (1.15)
13	3594	2.00 (1.09)	1.99 (1.12)
14	3347	2.07 (1.09)	2.05 (1.13)
15	2312	2.11 (1.05)	2.09 (1.09)
16	353	1.99 (1.07)	1.96 (1.12)
17	841	1.79 (0.97)	1.77 (1.00)
18	86	1.92 (1.05)	1.90 (1.09)
Gender			
Female	10946	1.95 (1.10)	1.93 (1.14)
Male	8814	1.80 (1.14)	1.76 (1.20)
School night bedtime			
Bedtime set by other	10425	1.75 (1.11)	1.71 (1.16)
Bedtime set by self	9335	2.03 (1.11)	2.01 (1.16)
Weekend bedtime			
Bedtime set by other	5397	1.40 (0.98)	1.36 (1.04)
Bedtime set by self	14363	2.07 (1.12)	2.04 (1.16)
Electronic media curfew			
Yes	10202	1.76 (1.10)	1.72 (1.15)
No	9558	2.02 (1.13)	1.99 (1.17)
Social media before sleep			
Never	2960	1.36 (1.06)	1.29 (1.14)
Rarely	1707	1.57 (1.04)	1.53 (1.09)
Sometimes	2290	1.68 (1.02)	1.65 (1.07)
Often	3064	1.84 (1.03)	1.81 (1.07)
Daily	9739	2.16 (1.12)	2.14 (1.16)
Video gaming before sleep			
Never	3745	1.67 (1.05)	1.64 (1.10)
Rarely	3511	1.69 (1.04)	1.65 (1.09)
Sometimes	3505	1.80 (1.05)	1.78 (1.08)
Often	3588	1.93 (1.11)	1.91 (1.15)
Daily	5411	2.18 (1.20)	2.13 (1.27)

Social jetlag is reported in decimal hours.

### Rules on Setting Bedtime, Rules on Electronic Media Use, and Frequency of Electronic Media Use (Social Media and Gaming) Before Sleep

As summarised in Table 1, 47.2% of participants reported that they set their own bedtime on a school night, while this rose to 72.7% at the weekend. The proportion of young people who reported that they did have a rule or set time in their house on a school night (51.6%) about when they were supposed to turn off or put away computers, phones or other electronics was similar to those who did not (48.4%). Approximately half of the participants (49.3%) reported that they used social media daily in the hour before they intended to sleep compared with just over a quarter (27.4%) who played video games daily in the hour before they intended to sleep. Descriptive statistics for social jetlag by bedtime setting, electronic media curfew, and frequency of electronic media use before sleep are presented in Table 2.

### Associations of Age, Gender, Bedtime Setting, Electronic Media Curfew, and Frequency of Electronic Media Use Before Sleep with Social Jetlag

A series of multiple regression models were performed to establish the extent to which age, gender, bedtime setting, electronic media curfew, and frequency of electronic media use before sleep could predict social jetlag. All regression models are presented in Table 3. As shown in Model 1, age (β = 0.13) and gender (β = −0.06) were associated with social jetlag and accounted for 2% of variance in social jetlag. Social jetlag was positively predicted by age: an increase in age was associated with higher social jetlag. Being male was associated with lower social jetlag than being female. Age and gender were included as covariates, and remained significant predictors of social jetlag, in all subsequent models. In Model 2, controlling for age and gender, school bedtime setting and weekend bedtime setting were both significant predictors of social jetlag. Setting your own bedtime on a school night was associated with lower social jetlag than when bedtime was set by another, but setting your own bedtime at the weekend was associated with higher social jetlag than when bedtime was set by another. After examining standardised beta values, weekend bedtime setting was a stronger predictor (β = 0.26) than school night bedtime setting (β = −0.03). All variables together accounted for 8% of variance in social jetlag. In Model 3, controlling for age and gender, not having an electronic media curfew on a school night was associated with higher social jetlag (β = 0.09) than if there was a rule or set time when they were supposed to turn off or put away computers, phones or other electronics. All variables together accounted for 3% of variance in social jetlag. In Model 4, controlling for age and gender, the frequency of social media use before sleep (β = 0.19) and the frequency of video gaming before sleep (β = 0.13) were positively associated with social jetlag. More frequent electronic media use was associated with higher social jetlag for both predictors, with all variables accounting for 9% of variance in social jetlag.

**Table 3.** Multiple regression analysis of age, gender, school night bedtime setting, weekend bedtime setting, electronic media curfew, social media use before sleep and video gaming before sleep as predictors of social jetlag (*n* = 19,760).

	*B*	SE *B*	β	*t*	*R*^*2*^	*p*
Model 1					.02	<.001
Age	0.08	0.00	0.13	18.81		<.001
Gender	−0.14	0.02	−0.06	−9.12		<.001
Model 2					.08	<.001
Age	0.04	0.00	0.06	8.06		<.001
Gender	−0.13	0.02	−0.06	−8.39		<.001
School bedtime setting	−0.07	0.02	−0.03	−3.96		<.001
Weekend bedtime setting	0.65	0.02	0.26	32.13		<.001
Model 3					.03	<.001
Age	0.06	0.00	0.11	15.17		<.001
Gender	−0.14	0.02	−0.06	−8.97		<.001
Electronic media curfew	0.19	0.02	0.09	11.89		<.001
Model 4					.09	<.001
Age	0.05	0.00	0.08	11.72		<.001
Gender	−0.17	0.02	−0.08	−10.73		<.001
Social media before sleep	0.15	0.01	0.19	25.15		<.001
Video gaming before sleep	0.10	0.00	0.13	17.33		<.001
Model 5					.12	<.001
Age	0.02	0.00	0.04	4.97		<.001
Gender	−0.16	0.02	−0.07	−10.16		<.001
School bedtime setting	−0.09	0.02	−0.04	−4.55		<.001
Weekend bedtime setting	0.52	0.02	0.21	25.76		<.001
Electronic media curfew	0.04	0.02	0.02	2.18		.03
Social media before sleep	0.12	0.01	0.16	20.27		<.001
Video gaming before sleep	0.09	0.01	0.12	15.64		<.001

*Note*. Gender coded 0 = female, 1 = male; Bedtime setting coded 0 = set by other, 1 = set by self; Electronic media curfew coded 0 = yes, 1 = no. For electronic media before sleep, higher scores indicate more frequent use (0–4). β = standardised beta coefficients.

All predictors were included, and remained significantly associated with social jetlag in Model 5, except for whether participants reported having an electronic media curfew (*p* = 0.03). School night bedtime setting (β = −0.04), weekend bedtime setting (β = 0.21) and the frequency of social media use before sleep (β = 0.16) and video gaming before sleep (β = 0.12) were associated with social jetlag. Setting your own bedtime on a school night remained associated with lower social jetlag and setting your own bedtime at the weekend remained associated with higher social jetlag. More frequent electronic media use before sleep remained associated with higher social jetlag. The final model accounted for 12% of variance in social jetlag. Results of regression analysis for actual social jetlag – includes negative social jetlag in cases when midsleep times on school days are later than on weekends – demonstrated a similar pattern to the regression results for social jetlag (absolute) presented here. Please see Table S3 in supplementary material.

## Discussion

This study provides novel evidence on the amount of social jetlag experienced by school-aged children and adolescents in a large community sample in England, as well as insights into relations between social jetlag and rule-setting around bedtime and electronic media use as well as frequency of electronic media use before sleep. Social jetlag was common in young people aged 9–18 y, with an average social jetlag of 1.88 h (1 hr, 53 min). As expected, we found that social jetlag was positively associated with age. Based on visual exploration of social jetlag by age, social jetlag appears to have increased from childhood – specifically those in later primary school years – to peak at age 15, with an approximate mean difference in social jetlag of 35 min between these years. After this peak, social jetlag appears to decrease in the older age groups (16–18 y). This supports findings presented by Randler and colleagues suggesting that the ages of 16–17 y represent a “breakpoint” where sleep behaviour changes back towards less jetlagged behaviour (Randler et al. 2019). Social jetlag was associated with gender, so that being female, compared with being male, was associated with higher social jetlag. While females were found to have significantly greater social jetlag than males, our findings indicated only a small mean difference of 9 min between girls (1.95 h: 1 hr, 57 min) and boys (1.80 h: 1 hr, 48 min), suggesting that this difference may not be practically meaningful. The association found between social jetlag and gender with school-aged students is consistent with phase delay (when circadian timing shifts later) being puberty-related, and with evidence that girls begin to delay sleep timing one year earlier than boys and show maximum eveningness at a younger age (girls: 15–19.5 y; boys: 18–21 y) (Hagenauer and Lee 2013; Roenneberg et al. 2004). Therefore, girls in this sample may have been more likely to delay bedtimes at the weekend and to have done so at an earlier age, contributing to higher social jetlag. Furthermore, gender role expectations – for example, morning grooming routines or more household chores – may increase the likelihood that girls wake earlier than boys on weekdays (Fredriksen et al. 2004).

Regarding autonomy, we found that the presence of rules around bedtime setting at the weekend were associated with lower levels of social jetlag as predicted. In fact, whether a rule was in place at the weekend emerged as the strongest predictor of social jetlag. In the final model, when all other predictors were held constant, social jetlag increased by 0.52 h (31.2 min) if an individual set their own bedtime compared to someone whose bedtime was set by another. Given the absence of an early school start the next day, it is perhaps unsurprising that 72.7% of young people set their own bedtime at the weekend compared with 47.2% on a school night. Young people may stay up later and wake up later when the opportunity arises at the weekend, partly demonstrating delayed sleep-wake timing that reflects changes to the biological regulation of sleep during adolescence (Crowley et al. 2018; Gradisar et al. 2011). Reduced societal demands at the weekend, specifically relating to days when students are not required to get up in time for school, increase this likelihood, although some students might continue to have fixed early morning commitments on weekends, such as sporting activities, possibly explaining the handful who had negative social jetlag. Many students may be able to sleep at the weekend for a more desired amount of time rather than having their sleep restricted by the start of the school day. Our finding is in line with that of a study in Finland, where adolescents whose main reason for going to bed on weekends was parent-set bedtimes, went to bed earlier and had an earlier midpoint of sleep than other groups (Short et al. 2019). Insufficient sleep during the school week may mean that sleep duration is longer at the weekend and lie-ins commonplace (Owens et al. 2014), which could in turn contribute to greater social jetlag. However, it should be noted that social jetlag and associated outcomes are a consequence of constraints imposed by social clocks on work days – which we might extrapolate to school days for students – rather than sleeping in on free days in an attempt to catch-up on sleep (Roenneberg et al. 2019). This study did not include an item on whether any rules were in place regarding rise times at the weekend, or whether alarms were being used to wake up, but their absence may also potentially contribute to the amount of social jetlag reported. Our findings provide initial support for the beneficial role of parents/carers or other family members in setting bedtimes at the weekend to help reduce sleep irregularity and limit social jetlag. Although, it could be that monitoring weekend bedtimes might be challenging if, for example, those setting the time might go to bed earlier than the adolescent.

Contrary to our predictions, findings regarding school nights were in the opposite direction, and setting your own bedtime on a school night was associated with lower social jetlag than when bedtime was set by another. Considering the final model, social jetlag decreased by 0.09 h (5.4 min) if an individual set their own bedtime on a school night compared to someone whose bedtime was set by another on a school night. Although we acknowledge that the small association found in our study means it seems unlikely that whether you set your own bedtime or not on a school night would actually be impactful in everyday life, given the amount of social jetlag experienced, this warrants further discussion as it contrasts with findings over weekend bedtime setting. As a potential explanation, when a young person has to get up in time for school but does not have a parent-set bedtime on a school night – previously found to relate to later bedtimes (Short et al. 2011) – then a later bedtime might contribute to a later midpoint of sleep. Any discrepancy between midsleep on a school night and midsleep at the weekend could thereby be reduced, lessening social jetlag. Another possible explanation may be that those who naturally go to bed later at the weekend, could also be those who have bedtimes set by others on a school night to try to limit late bedtimes.

Students were asked whether they had a household rule or set time about when they were supposed to turn off or put away computers, phones or other electronics on school nights. Although we hypothesised that rules around electronic media use would be associated with lower levels of social jetlag, in the final model, a curfew regarding electronic media use did not significantly predict social jetlag. When considering possible explanations, it is plausible that even if a rule were present, this may not have been sufficiently early to impact on the young person’s sleep onset time or that they habitually adhered to this curfew. We did not ask about rules over electronic media at the weekend and it may be informative in future work to investigate whether their presence is associated with social jetlag, especially given our finding regarding bedtime setting at the weekend. It is unclear what the impact of limits on electronic device use might be at the weekend as this was not asked specifically. If young people are not set limits over device use at the weekend, it could be proposed that sleep onset and offset may be even later with a corresponding later midpoint of sleep and increased social jetlag.

Both frequency of social media use and frequency of video gaming before sleep intention were associated with social jetlag, and emerged as the most important predictors after rules on weekend bedtime setting. As predicted, high frequency of social media use and high frequency of video gaming in the hour before students intended to go to sleep were related to higher levels of social jetlag. This is in line with findings from an adult sample, where frequent (more than a few times a week) internet use in the hour before bed was associated with social jetlag in Australia (Lang et al. 2018). The measures included in our study focused on what happened in the hour before sleep rather than before bedtime. Young people might continue to use electronic media after they have gone to bed, and so asking about behaviour before sleep intention could be a more nuanced approach to sleep measurement than asking only about behaviour before going to bed, which may well be how “bedtime” is interpreted. For example, they could be going to bed to catch-up with friends and not be intending to sleep for a while. Although observational studies have demonstrated a robust inverse relationship between electronic media use and sleep outcomes, evidence on the proposed mechanisms underlying these associations is less clear (Hale et al. 2019). It is not known why those young people in our study who were using social media or gaming before sleep on a regular basis have greater social jetlag. It could be that they are individuals with later chronotypes, or perhaps the use of electronic media may support the “sleep displacement hypothesis” (Bauducco et al. 2024), whereby the time spent using technology replaces time that might have been spent sleeping with a delayed sleep onset at the weekend.

### Limitations and Future Directions

Study findings should be considered within the context of its limitations. A high proportion (99.8%) demonstrated social jetlag, which is higher than that recorded with a sample of 12- to 19-year-olds in India (73.9%) (Chandrakar 2017). It should be noted that this high percentage, alongside our finding that only 32 students demonstrated zero jetlag, may be influenced in part by the survey’s use of a sliding scale to measure sleep timing. The method of measurement used in this study may have reduced respondents’ ability, if desired, to provide consistent times for weekend and school night sleep/wake timing, therefore differences between the two midpoints of sleep and hence social jetlag were more likely to arise. We did not investigate chronotype as items necessary to calculate this measure (Roenneberg et al. 2012, 2019) were not collected as part of the OxWell Student Survey. Therefore, we are unable to comment on whether the peak of social jetlag coincided with the peak of chronotype in this sample. This study was cross-sectional which limits our ability to make inferences about social jetlag with increasing age or causal links between predictors and social jetlag. Furthermore, our data do not contribute enough detail to enable the assessment of social jetlag with those who did not identify as a binary gender, and subsequent research with gender diverse students is recommended. Lastly, although we consider it a strength of the study that we focused on types of electronic device activity before sleep that often require active engagement – and these data are beneficial in mapping young people’s usage of social media and gaming in a large sample – we acknowledge that our study did not include an item about general electronic device use before sleep. Consequently, we are not able to investigate whether the use of electronic devices more broadly would be a predictor of social jetlag. Future studies looking at longitudinal patterns could help to further our understanding of changes in social jetlag over time and elucidate associations between social jetlag, autonomy and electronic media use across child and adolescent development.

This study provides an initial insight into modifiable factors that may assist in curbing social jetlag in young people. Based on our findings, three strategies are proposed with the potential to lessen social jetlag: support young people to set bedtimes at the weekend; reduce social media use in the hour before sleep intention; and reduce gaming in the hour before sleep intention. Experimental work is needed utilising home-based protocols to manipulate household rules concerning bedtimes, as well as the frequency of social media use and gaming before sleep, to assess any impact on school day and weekend sleep/wake timing and social jetlag. Our study was not designed to examine associations between social jetlag and wellbeing in young people, but a greater understanding of these links may provide a rationale to help families and young people put bedtimes in place consistently across the week and establish limits on social media use and gaming before sleep. The main future direction proposed is to examine associations between social jetlag and mental health, in particular symptoms of anxiety and depression, as well as school factors, such as school attendance.

## Conclusions

Social jetlag peaks at the age of 15, with a difference of approximately two hours between school days and weekends. Weekend bedtime setting, and frequency of social media use and video gaming before sleep, were most strongly associated with social jetlag. Encouragingly, for children and adolescents experiencing social jetlag, our findings suggest that family decisions on weekend bedtime setting as well as the young person’s electronic media use before sleep have the potential to contribute to reduced social jetlag and more regular school night/weekend sleep timing, with consequent benefits to young people.

## Supplementary Material

Supplemental Material

## Data Availability

Fully deidentified extracts of the data can be provided to academic research collaborators upon reasonable request after a review process by the research team to ensure that uses of the data fall under the remit of the intended purposes set out in the privacy information and to prevent duplication of analyses. The data are not publicly available because of ethical and information governance restrictions. The full list of questions as well as other details are available on a project-specific OxWell Open Science Framework website along with the study protocol (https://osf.io/sekhr/). Full data dictionaries can be made available upon approval for access to data extracts.
