# Supplementary material for: Social jetlag and sleep habits in children and adolescents: Associations with autonomy (bedtime setting and electronics curfew) and electronic media use before sleep
Source: Chronobiol Int. 2025 Jan 6;42(1):46–57. doi: 10.1080/07420528.2024.2444675 (PMC11854036; doi:10.1080/07420528.2024.2444675)
Supplement: Supplemental Material [file ICBI_A_2444675_SM2448.pdf]

Supplementary material

Table S1. Sample characteristics for students who provided gender as female or male (*n* = 22,294), those who did not provide binary gender (*n* = 1205), and overall sample (*n* = 23,499)

	Gender (binary) (*n* = 22,294)	Gender (other, prefer not to answer, missing) (*n* = 1205)	Overall (*n* = 23,499)
Age (years)			
Mean (SD)	12.7 (1.93)	12.9 (1.83)	12.7 (1.93)
Gender, n (%)			
Female	12212 (54.8%)	0 (0%)	12212 (52.0%)
Male	10082 (45.2%)	0 (0%)	10082 (42.9%)
Missing	0 (0%)	1205 (100%)	1205 (5.1%)
Social jetlag (hrs)			
Mean (SD)	1.89 (1.12)	2.06 (1.24)	1.90 (1.13)
Actual social jetlag (hrs)			
Mean (SD)	1.85 (1.18)	2.01 (1.33)	1.86 (1.19)
School bedtime setting, n (%)			
Bedtime set by other	11551 (51.8%)	543 (45.1%)	12094 (51.5%)
Bedtime set by self	10244 (45.9%)	616 (51.1%)	10860 (46.2%)
Missing	499 (2.2%)	46 (3.8%)	545 (2.3%)
Weekend bedtime setting, n (%)			
Bedtime set by other	5973 (26.8%)	277 (23.0%)	6250 (26.6%)
Bedtime set by self	15725 (70.5%)	877 (72.8%)	16602 (70.6%)
Missing	596 (2.7%)	51 (4.2%)	647 (2.8%)
Electronic media curfew, n (%)			
Yes	10898 (48.9%)	560 (46.5%)	11458 (48.8%)
No	10133 (45.5%)	543 (45.1%)	10676 (45.4%)
Missing	1263 (5.7%)	102 (8.5%)	1365 (5.8%)
Social media use before sleep, n (%)			
Never	3265 (14.6%)	141 (11.7%)	3406 (14.5%)
Rarely	1919 (8.6%)	85 (7.1%)	2004 (8.5%)
Sometimes	2577 (11.6%)	116 (9.6%)	2693 (11.5%)
Often	3401 (15.3%)	178 (14.8%)	3579 (15.2%)
Daily	10700 (48.0%)	662 (54.9%)	11362 (48.4%)
Missing	432 (1.9%)	23 (1.9%)	455 (1.9%)
Mean (SD)	2.75 (1.50)	2.96 (1.42)	2.76 (1.49)
Video gaming before sleep, n (%)			
Never	4030 (18.1%)	152 (12.6%)	4182 (17.8%)
Rarely	3840 (17.2%)	174 (14.4%)	4014 (17.1%)
Sometimes	3842 (17.2%)	176 (14.6%)	4018 (17.1%)
Often	3940 (17.7%)	252 (20.9%)	4192 (17.8%)
Daily	5972 (26.8%)	409 (33.9%)	6381 (27.2%)
Missing	670 (3.0%)	42 (3.5%)	712 (3.0%)
Mean (SD)	2.18 (1.48)	2.51 (1.43)	2.20 (1.47)

Note. Social jetlag is reported in decimal hours. Electronic media before sleep is scored 0–4.

Table S2. Sample characteristics for students who provided social jetlag, age and gender (*n* = 22,294) with all predictor variables (*n* = 19,760) and missing one or more predictor variables (*n* = 2534)

	Social jetlag and all predictors (*n* = 19,760)	Social jetlag & missing >1 predictors (*n* = 2534)	Overall (*n* = 22,294)
Age (years), n (%)			
9	406 (2.1%)	68 (2.7%)	474 (2.1%)
10	2598 (13.1%)	395 (15.6%)	2993 (13.4%)
11	2699 (13.7%)	422 (16.7%)	3121 (14.0%)
12	3524 (17.8%)	496 (19.6%)	4020 (18.0%)
13	3594 (18.2%)	427 (16.9%)	4021 (18.0%)
14	3347 (16.9%)	388 (15.3%)	3735 (16.8%)
15	2312 (11.7%)	218 (8.6%)	2530 (11.3%)
16	353 (1.8%)	39 (1.5%)	392 (1.8%)
17	841 (4.3%)	69 (2.7%)	910 (4.1%)
18	86 (0.4%)	12 (0.5%)	98 (0.4%)
Mean (SD)	12.7 (1.94)	12.4 (1.88)	12.7 (1.93)
Gender, n (%)			
Female	10946 (55.4%)	1266 (50.0%)	12212 (54.8%)
Male	8814 (44.6%)	1268 (50.0%)	10082 (45.2%)
Social jetlag (hrs)			
Mean (SD)	1.88 (1.12)	1.90 (1.15)	1.89 (1.12)
Actual social jetlag (hrs)			
Mean (SD)	1.85 (1.17)	1.86 (1.22)	1.85 (1.18)
School bedtime setting, n (%)			
Bedtime set by other	10425 (52.8%)	1126 (44.4%)	11551 (51.8%)
Bedtime set by self	9335 (47.2%)	909 (35.9%)	10244 (45.9%)
Missing	0 (0%)	499 (19.7%)	499 (2.2%)
Weekend bedtime setting, n (%)			
Bedtime set by other	5397 (27.3%)	576 (22.7%)	5973 (26.8%)
Bedtime set by self	14363 (72.7%)	1362 (53.7%)	15725 (70.5%)
Missing	0 (0%)	596 (23.5%)	596 (2.7%)
Electronic media curfew, n (%)			
Yes	10202 (51.6%)	696 (27.5%)	10898 (48.9%)
No	9558 (48.4%)	575 (22.7%)	10133 (45.5%)
Missing	0 (0%)	1263 (49.8%)	1263 (5.7%)
Social media use before sleep, n (%)			
Never	2960 (15.0%)	305 (12.0%)	3265 (14.6%)
Rarely	1707 (8.6%)	212 (8.4%)	1919 (8.6%)
Sometimes	2290 (11.6%)	287 (11.3%)	2577 (11.6%)
Often	3064 (15.5%)	337 (13.3%)	3401 (15.3%)
Daily	9739 (49.3%)	961 (37.9%)	10700 (48.0%)
Missing	0 (0%)	432 (17.0%)	432 (1.9%)
Mean (SD)	2.75 (1.50)	2.68 (1.48)	2.75 (1.50)
Video gaming before sleep, n (%)			
Never	3745 (19.0%)	285 (11.2%)	4030 (18.1%)

Rarely	3511 (17.8%)	329 (13.0%)	3840 (17.2%)
Sometimes	3505 (17.7%)	337 (13.3%)	3842 (17.2%)
Often	3588 (18.2%)	352 (13.9%)	3940 (17.7%)
Daily	5411 (27.4%)	561 (22.1%)	5972 (26.8%)
Missing	0 (0%)	670 (26.4%)	670 (3.0%)
Mean (SD)	2.17 (1.48)	2.31 (1.44)	2.18 (1.48)

Note. Social jetlag is reported in decimal hours. Electronic media before sleep is scored 0–4.

Table S3. Multiple regression analysis of age, gender, school night bedtime setting, weekend bedtime setting, electronic media curfew, social media use before sleep and video gaming before sleep as predictors of actual social jetlag (*n* = 19,760)

	B	SE *B*	β	*t*	R^2	*p*
Model 1					.02	<.001
Age	0.08	0.00	0.14	19.86		<.001
Gender	-0.17	0.02	-0.07	-9.98		<.001
Model 2					.08	<.001
Age	0.04	0.00	0.07	9.37		<.001
Gender	-0.15	0.02	-0.06	-9.30		<.001
School bedtime setting	-0.08	0.02	-0.04	-4.20		<.001
Weekend bedtime setting	0.66	0.02	0.25	31.38		<.001
Model 3					.03	<.001
Age	0.07	0.00	0.12	16.29		<.001
Gender	-0.16	0.02	-0.07	-9.84		<.001
Electronic media curfew	0.19	0.02	0.08	11.47		<.001
Model 4					.09	<.001
Age	0.05	0.00	0.09	12.53		<.001
Gender	-0.19	0.02	-0.08	-11.27		<.001
Social media before sleep	0.15	0.01	0.20	25.44		<.001
Video gaming before sleep	0.10	0.01	0.12	16.07		<.001
Model 5					.12	<.001
Age	0.03	0.00	0.05	6.04		<.001
Gender	-0.18	0.02	-0.07	-10.72		<.001
School bedtime setting	-0.09	0.02	-0.04	-4.78		<.001
Weekend bedtime setting	0.53	0.02	0.20	25.08		<.001
Electronic media curfew	0.03	0.02	0.01	2.00		.046
Social media before sleep	0.13	0.01	0.16	20.71		<.001
Video gaming before sleep	0.08	0.01	0.11	14.40		<.001

Note. Gender coded 0 = female, 1 = male; Bedtime setting coded 0 = set by other, 1 = set by self; Electronic media curfew coded 0 = yes, 1 = no. For electronic media before sleep, higher scores indicate more frequent use (0–4). β = standardised beta coefficients.
